# Biodegradable cellulose nanocrystals hydrogels for removal of acid red 8 dye from aqueous solutions

**DOI:** 10.1038/s41598-022-10087-1

**Published:** 2022-04-19

**Authors:** Radwa Mohamed Abdelaziz, Azza El-Maghraby, Wagih Abdel-Alim Sadik, Abdel-Ghaffar Maghraby El-Demerdash, Eman Aly Fadl

**Affiliations:** 1 Department of Materials Science, Institute of Graduate Studies and Research, Alexandria University, 163 Horreya Avenue, Alshatby, 21526 Alexandria Egypt; 2 Fabrication Technology Research Department, Advanced Technology and New Materials Research Institute (ATNMRI), City of Scientific Research and Technological Applications (SRTA-City), New Borg El Arab, Egypt

**Keywords:** Pollution remediation, Nanoscale materials

## Abstract

Biodegradable cellulose nanocrystals hydrogels (CNCsH) were synthesized from cellulose nanocrystals (CNCs) which were prepared from office wastepaper (OWP) by a chemical crosslinking method using epicholorohydrin (ECH) as a cross-linker. CNCsH were tested for their swelling behavior and biodegradability and the point of zero charge had been determined. The ability of CNCsH for removing the Acid Red 8 (AR8) anionic dye from its aqueous solution was evaluated. The different parameters affecting removal of the dye, such as pH, initial concentration of dye, content of CNCs, temperature and adsorbent dosage were investigated. The optimum conditions for 68% removal efficiency were pH = 1, initial concentration of dye = 10 ppm, contact time = 105 min, CNCs content = 5% and CNCsH dosage = 0.5 g at 30 °C. The adsorption isotherms, kinetics, and thermodynamic parameters have been studied. The results showed an appropriate fit for Langmuir adsorption isotherm and pseudo-second order kinetics model with an adsorption capacity of 17.12 mg/g. According to the obtained values of thermodynamic parameters, the removal of Acid red 8 by CNCs hydrogels was exothermic spontaneous process.

## Introduction

As a result of industrialization and urbanization, an enormous amount of industrial wastewater and domestic sewage are produced causing an increase in water pollution. This increase not only causes dangerous diseases but also creates deterioration in the ecosystem of water bodies leading to impeding economic development in the long run^1,2^. Today, Water pollution by textile industries effluents has become one of the most serious environmental problems as they release a wide variety of pollutants such as dyes, soap, enzymes, complex compounds, and heavy metals^3,4^. Dyes are one of the main pollutants released from the textile industry, their degradation products are carcinogenic and can pose serious health problems to humans including dysfunction of kidney, eye irritation, and allergic disease. Moreover, dyes cause aesthetic pollution of the environment^5–7^. Because of their high solubility and visibility in water bodies, they minimize the amount of sunlight penetrating the water, resulting in a decrease in the photosynthesis process and a deficiency in the concentration of dissolved oxygen^8,9^. Dyes can classify according to their origin, solubility, or particle charge, but classifying based on their chemical nature (acid (anionic), base (cationic), direct, disperses, reactive, sulfur, and vat dyes) is the renowned one^10^. Anionic dyes, containing negatively charged sulfonic groups (SO_3_^−^H^+^), are utilized commonly in textile dyeing industries. Techniques like membrane separation, electrocoagulation, advanced oxidation processes, adsorption, and photochemical degradation are used for removing anionic dye from industrial effluent^11–14^. Treatment of industrial wastewater by adsorption has been proven to be an efficient and low-cost technique amongst these techniques^15,16^. Different types of adsorbents have been used to remove dyes, including activated carbon, bio-adsorbent, nanoparticles, fly ash, and carbon nanotubes^17,18^.

Even though various adsorbents, hydrogels, 3D networks of cross-linked polymer, are recognized as effective adsorbents owning to their high adsorption capacity, low cost, and easy operation^19,20^. However, during the past few decades, polysaccharide-based hydrogels have attracted significant interest attributing to their ability to remove dyes from wastewater without generating secondary pollution because of their biodegradability^21^. Among numerous polysaccharide materials, cellulose is deemed as the most prevalent polysaccharide polymer in nature which could be isolated from agricultural and industrial wastes, such as wheat straw, rice husk, soy hull and oil palm empty fruit bunches^22^. Cellulose consists of β-D-glucopyranose units linked together β-1,4-glycosidic linkage. Hydroxyl groups, which exist along the backbone of cellulose molecules, act as active sites for dye adsorption^23^.

Although wastepaper is considered a problematic waste, it can also be consider as an attractive cellulosic resource for producing of cellulose because it is abundant with low cost^24^. Cellulose molecules are aggregated into microfibrils which are composed of both ordered, (crystalline phase) and disordered, called (amorphous phase)^25,26^. Cellulose nanocrystals (CNCs), a crystalline rod—shaped particles, are traditionally obtained by acid hydrolysis process which dissolve the amorphous parts in the cellulose fibrils, leaving the crystalline parts freed^27^. Few papers reported the extraction of CNCs from office waste paper (OWP). Lei et al.^28^ have successively prepared CNCs from OWP through cooperating the chemical agents and the mechanical forces by using deinking agents and laboratory repulper, pursued by an acid hydrolysis. CNCs have recently achieved a considerable interest because of their abundance, degradability and high specific surface area^29^.

To date, preparation of hydrogels based on CNCs depend on physical or chemical incorporation of CNCs within polymeric hydrogel networks^30^ such as polyacrylamide^31^, alginate^32^ and carboxymethylated chitosan^33^. It had been rarely reported CNCs alone as single-component gels due to its limited ability to entangle^30^.

The aim of this work is to prepare CNCs from office wastepaper and use it for preparing single component CNCsH to be used for removing AR8 dye from its aqueous solutions. CNCsH was fabricated by chemically crosslinking different concentration of the prepared CNCs using epicholorohydrin (ECH) as cross linker. The extracted cellulose and the prepared CNCs and CNCs hydrogels were characterized with various characterization tools. Factors affecting the adsorption of AR8 dye on hydrogel such as pH, contact time, hydrogel dosage, dye initial concentration, CNCs content and temperature were studied. Isotherms, kinetics and thermodynamic studies for the adsorption process were investigated. Moreover, swelling behavior, biodegradability and reusability along with point of zero charge of the CNCsH were also investigated.

## Materials and methods

### Materials

Office wastepaper (OWP) was used as a raw material in this study. Sodium hydroxide (NaOH) (98%), Sulfuric acid (H_2_SO_4_) (95–97%) and Hydrochloric acid (HCl) (36.5–38%) were supplied by Fischer scientific Company (USA). Hydrogen peroxide (H_2_O_2_) (50%) and Urea (CH_4_N_2_O) were Purchased from Piochem (Egypt). Ammonium sulphate ((NH_4_)_2_SO_4_) (99%), zinc chloride (ZnCl_2_) (98%) and Dipotassium hydrogen phosphate (K_2_HPO_4_) (98%) were received from ElNasr company (Egypt). Acid red 8 (C_18_H_14_N_2_Na_2_O_7_S_2_) was received from Chemajet company (Egypt). Epichlorohydrin (ECH) (C_3_H_5_ClO) (98.5%) was supplied by Lobachemie company (India). All chemicals were used as received without further treatment.

### Methods

#### Extraction of cellulose from office wastepaper

50 g of (OWP) were cut into small pieces by paper shredder and immersed in hot water then filtered and kept at room temperature to dry. After drying, the sample was grounded by high speed blender. The extraction process of cellulose takes place in three steps. The first step includes treatment of grounded wastepaper with (12%) NaOH solution at 25 °C for 24 h to swell cellulose fibers, as well as to remove the ink residual particles and hemicelluloses. The second step is to immerse the treated cellulose for 2 h in (1.0 M) HCl solution at 80 °C to remove the lignin residuals. The third step is to immerse the pretreated cellulose at 60 °C for 2h in a mixture of (20%) H_2_O_2_ and (5%) NaOH. After each step, cycles of filtration and washing was carried for the mixture till the pH becomes neutral.

#### Preparation of cellulose nanocrystals

5 g of the extracted cellulose were mixed with 50 ml of (47%) H_2_SO_4_ and stirred for 1 h at 45 °C. 100 ml of cold water was added to quench the acid hydrolysis process, followed by centrifugation to separate the prepared CNCs. Several washing cycles were carried until neutral pH was obtained.

#### Preparation of cellulose nanocrystals hydrogel

In order to dissolve cellulose, a solution was prepared from NaOH/urea/H_2_O with the ratio 7:12:81 to be used as solvent^34^. The desired amount of CNCs powder was dispersed in 100 ml of the prepared solvent and stirred for 30 min, followed by storing the CNCs solution at – 12 °C for 15 h. Lastly, a translucent CNCs solution was obtained by stirring the frozen solution for 10 min at 25 °C. The hydrogel was prepared by adding 6 ml ECH, as a crosslinking agent, drop wise to the CNCs solutions. The resulting mixture, after complete ECH feeding, was stirred at 25 °C for 1h to obtain a homogenized solution, then its transferred directly into 50 ml beaker and heated at 60 °C for 2 h. The prepared CNCsH were soaked in water for 24 h in order to eliminate any remaining residues and then dried at 60 °C. A series of CNCsH were obtained with various CNCs weight contents (4, 5, 6%). The resultant hydrogels were labelled as (4, 5 and 6%).

## Characterization

### Chemical structure analysis

The chemical structure of OWP, extracted cellulose, prepared CNCs and CNCsH were characterized by using Fourier Transform Infrared Spectroscopy (Spectrum BX 11 Infrared spectrometer FTIR LX 18-5255 Perkin Elmer).

### Morphology analysis

Scanning electron microscopy (SEM "JL JSM 6360LA", Japan) was used for investigating the morphology of OWP, extracted cellulose and prepared CNCsH. The particle shape and size of CNCs were observed using the Transmission electron microscopy (TEM “JEOL 2100PLUS”, Japan).

### Crystallinity analysis

The crystallinity of extracted cellulose and prepared CNCs were studied by using X-ray diffraction (XRD) patterns (X-ray 7000 Shimadzu-Japan). The degree of crystallinity (CI %) of the samples was calculated using Seagal’s method^35^ using Eq. (1).

1 $${\text{CI }}\left( \% \right) = \frac{{{\text{I}}_{{{\text{crystalline}}}} - {\text{I}}_{{{\text{amorphous}}}}}}{{{\text{I}}_{{{\text{crystalline}}}}}} \times 100$$

where I_crystalline_ = Intensity of crystalline peak and I_amorphous_ = Intensity of non-crystalline peak.

## Determination of pH of point of zero charge for the prepared CNCsH (pH _PZC_)

The pH of point of zero charge (pH_pzc_) of the hydrogel was determined according to the pH drift equilibrium technique^36^. A stock solution of (0.01 M) NaCl was prepared and 50 ml portions were transferred into 10 separate 250 ml flasks. The pH of the solutions was altered between 1 and 10 by adding NaOH/HCl such that each flask had a different pH value. A known amount of the hydrogel (0.1 g) was added to each solution. The solutions were equilibrated for 48 h before measuring the final pH. A graph of final pH verses the initial pH was plotted and the point of intersection is the point taken as pH_pzc_ of the hydrogel.

## Swelling studies

A pre-weighed 10 mg of the prepared CNCsH were placed in 100 ml of distilled water for two days at 25 °C. The Weight of the swollen CNCsH was measured after the excess surface water was blotted with filter paper superficially. The swelling ratio was determined using Eq. (2)^37^.

2 $${\text{Swelling ratio}} \; \left( \% \right) = \frac{{{\text{m}}_{{\text{t}}} - {\text{m}}_{{\text{o}}}}}{{{\text{m}}_{0}}} \times 100$$

where m_o_ is the mass of the dried CNCsH and m_t_ is the mass of the swollen the CNCsH.

## Biodegradability test

The biodegradability of CNCsH were evaluated by immersing 0.25 g of the prepared hydrogels in 100 ml of soil extraction solution for 10 days of incubation under room temperature. After10 days, the residue of the CNCsH was filtered out and dried at 60 °C in the oven for 4 h. Equation (3) was used to determine the weight loss of the CNCsH. The soil extract solution was prepared by immersing 250 g of soil in 250 ml of saline solution and the mixture was mixed for 2 h, then it was filtered to separate the soil particles. The filtered solution (30 ml) and the activation minerals (1.5 g of ammonium sulphate, 0.6 g of dipotassium hydrogen phosphate and 0.6 g of zinc chloride) were added to 270 ml of distilled water^38^.

3 $${\text{Weight loss}} \; \left( \% \right) = \frac{{{\text{m}}_{{\text{o}}} - {\text{m}}_{{\text{t}}}}}{{{\text{m}}_{{\text{o}}}}} \times 100$$

where m_0_ is the mass of dried CNCsH and m_t_ is the mass of CNCsH after incubation.

## Dye adsorption studies

The adsorption experiments of AR8 dye were carried out as follows: a stock solution of AR8 dye (1000 mg/L) was prepared. Different doses of dried CNCsH (0.1, 0.2, 0.3, 0.4, 0.5, 0.6 and 0.7 g) were immersed in 100 mls of AR8 dye solutions, which had been prepared with initial concentrations ranging between 10 and 30 ppm. The pH of the medium was adjusted by HCl and NaOH (1.0 M) to obtain final values (1, 2, 3, 5, 7 and 9). The adsorption process carried out in a shaking water bath at different temperatures (30, 40 and 50 °C), different shaking rate (100, 150, and 200 rpm) and different CNCs weight contents hydrogels (4, 5 and 6%). For analysis, samples were collected at different intervals. The concentration of the dye in the solution after the adsorption process was measured at (508 nm) by the UV−visible spectrophotometer. The percent of AR8 removal was calculated by Eq. (4), while the adsorption capacity was calculated by Eq. (5)^39^.

4 $${\text{Dye removal }}\left( \% \right) = \frac{{\left( {C_{o} - C_{t}} \right)}}{{C_{o}}} \times 100$$

5 $${\text{Adsorption capacity }}(q_{t} ){ } = \frac{{\left( {C_{o} {-}{ }C_{t} } \right){\text{V}}}}{W}{ }$$

where C_o_ is the initial concentration of AR8 (ppm), C_t_ is the final concentration of AR8 dye time t (ppm), V is the solution volume of AR8 (L), and W is the weight of the CNCsH (g), q_t_ is the adsorption capacity (mg/g) at time t. Isotherms, kinetics and thermodynamic studies for the adsorption process were also investigated.

## Reusability study

The reusability study was investigated at 30 °C by keeping 5% CNCs content hydrogel (0.5 g) in contact with AR8 dye solution (C_o_ = 10 ppm, V = 100 mL and pH = 1) for 105 minutes and under shaking speed of 150 rpm, then AR8- loaded CNCsH was filtrated and washed with distilled water, after that it was placed in 250 mL conical flask containing NaOH (1 M) for 105 min under shaking speed of 150 rpm. Then after, a solution of HCl (1 M) was used to neutralize CNCsH. The hydrogel was then collected, rinsed with distilled water and reused for the dye adsorption as stated above. To study the reusability of CNCsH, the adsorption–desorption cycle was conducted four times^40^.

## Results and discussion

### Mechanism of CNCs hydrogel preparation

**Figure 1:**
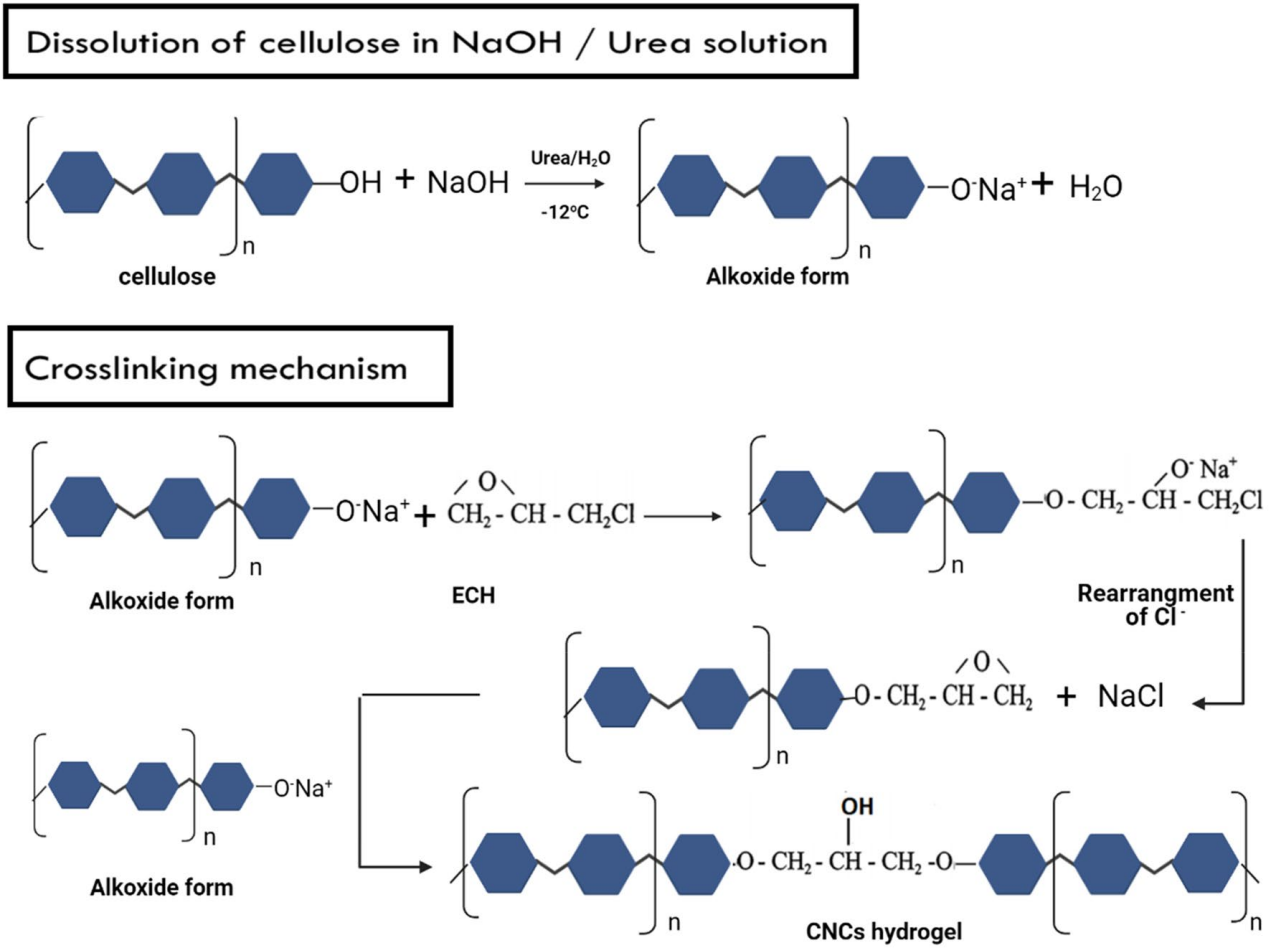
CNCsH preparation mechanism.

Dissolution of CNCs in NaOH /Urea / H_2_O solvent and the mechanism of CNCs crosslinking by cross linker ECH for preparation of hydrogel is shown in Fig. 1. In NaOH /Urea aqueous solution, CNCs dissolve and exist in a sodium alkoxide form. Based on williamson etherification and the alkali‐catalyzed oxalkylation, the alkoxide anion attacks the epoxy group of ECH forming monoether. A new epoxy group will yield from the rearrangement of chloride displacement. The new epoxy then will attack another alkoxide anion. So that the crosslinking reaction occur^41^.

### Characterization

#### Chemical structure analysis

**Figure 2:**
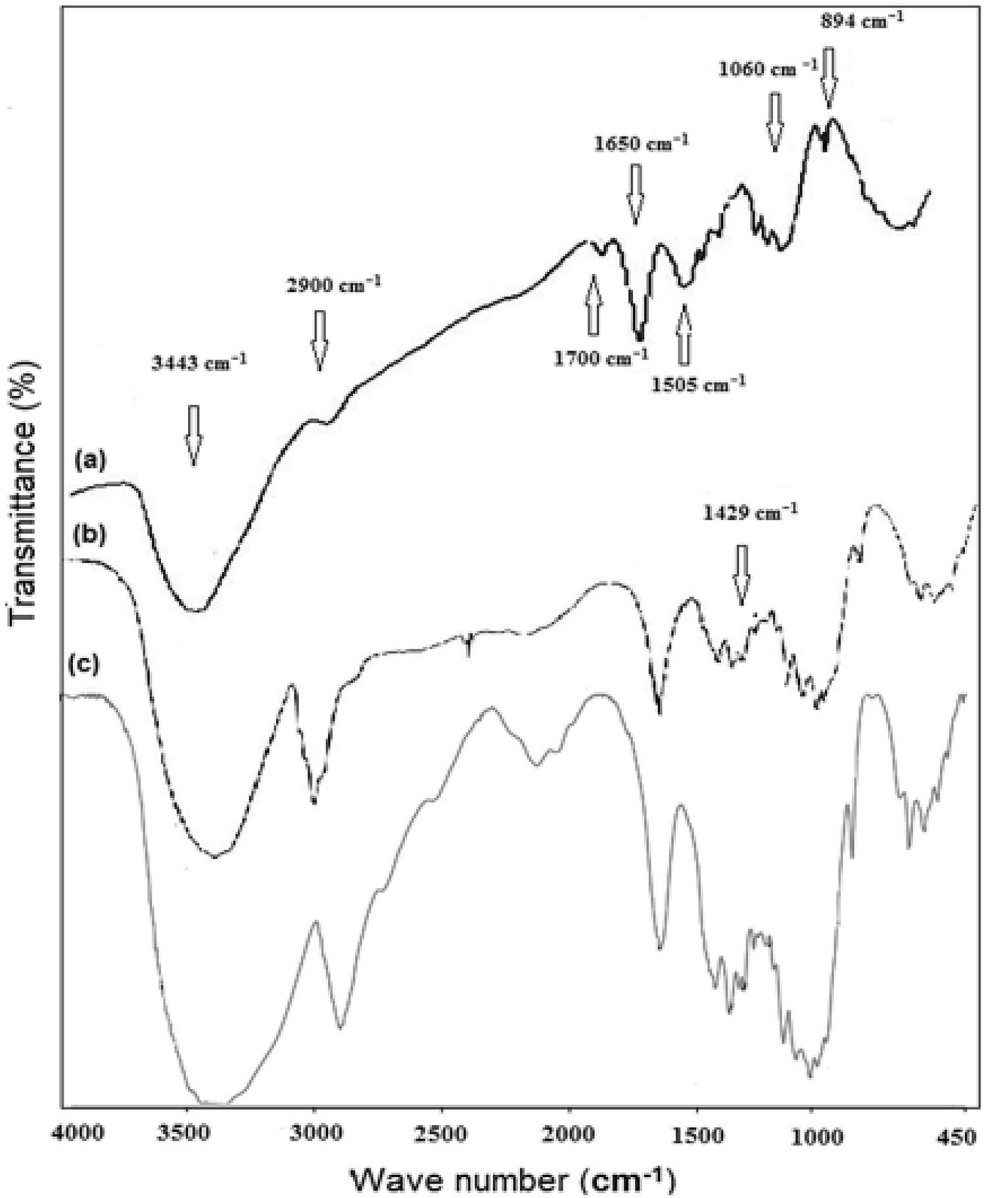
FTIR of (**a**) OWP, (**b**) Extracted cellulose and (**c**) CNCs.

**Figure 3:**
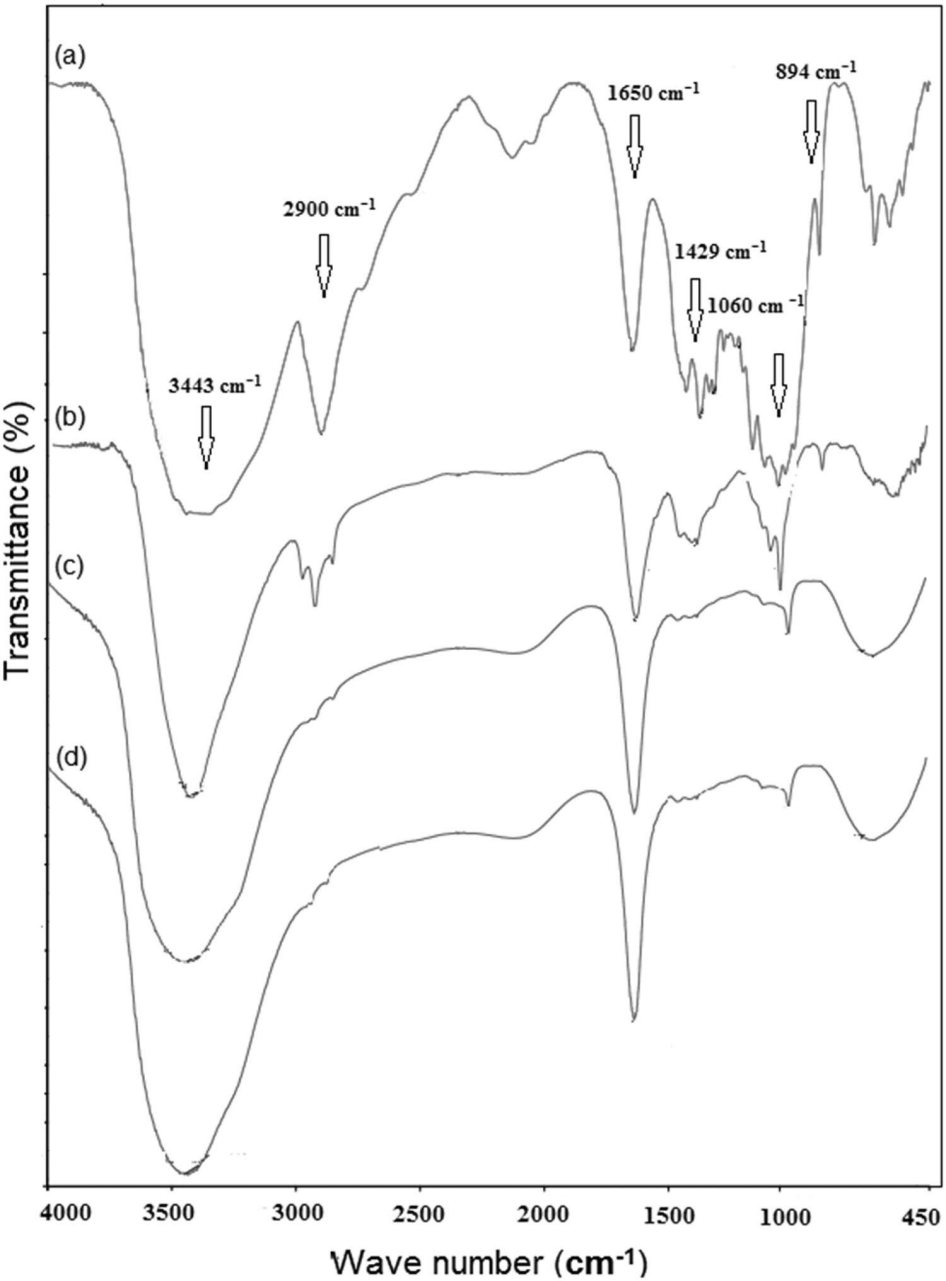
FTIR of (**a**) CNCs, (**b**) 4% CNCsH, (**c**) 5% CNCsH and (**d**) 6% CNCsH.

FTIR spectra of OWP, extracted cellulose, and CNCs are shown in Fig. 2 while in Fig. 3 spectra of CNCs and CNCsH are represented. The spectra of all samples showed the following peaks. A broad peak in the region from 3443 to 3344 cm^−1^ refers to the free O–H stretching vibration of the hydroxyl groups in cellulose molecules, while the peaks existing at 2900 cm^−1^ were due to C–H stretching vibration. Besides, two other peaks around 1060 cm^−1^ and 894 cm^−1^ confirm the presence of C–O–C pyranose ring stretching vibration and β-glycosidic linkage between glucose units, respectively^28,42^. Peak 3443 cm^−1^ has a higher intensity in the extracted cellulose spectrum than in the OWP spectrum, which is consistent with Ferre and Vila's conclusion that ink has an absorption band at 3380 cm^−1^ which causes a drop in OH group concentration^43^. In OWP spectrum, a peak around 1700 cm^−1^ refers to the stretching of C–O and a peak around 1505 cm^−1^ attributes to C in-plane aromatic vibrations are characteristic to hemicellulose and lignin, respectively. Their absence from extracted cellulose spectrum demonstrates the effectiveness of alkali and bleaching treatment^44^. By comparing the intensity of the peak at 1429 cm^−1^, known as “crystallinity band”, in the spectrum of CNCs with extracted cellulose spectrum, it was noticed that the CNCs has the higher crystallinity than extracted cellulose implying that the acidic hydrolysis process successively dissolves the amorphous regions in extracted cellulose and liberate CNCs. Compared with the CNCs spectrum, it was observed also that the intensity of the peak at 1429 cm^-1^ decreased in CNCsH ’s spectra, this may be because of the dissolution of the CNCs in the alkali solution during the hydrogel preparation. In contrast, the raise in the intensity of the peak at 1650 cm^−1^ known as “absorbed water peak” in CNCsH’s spectra compared to that of CNCs spectrum indicate that the CNCsH were more hydrophilic than CNCs, since the etherification reaction between ECH and CNCs stopped the adjacent CNCs chains from building intermolecular hydrogen bonds, thereby hydrophilic sites was introduced for water molecules to be absorbed^45,46^.

#### Morphology analysis

**Figure 4:**
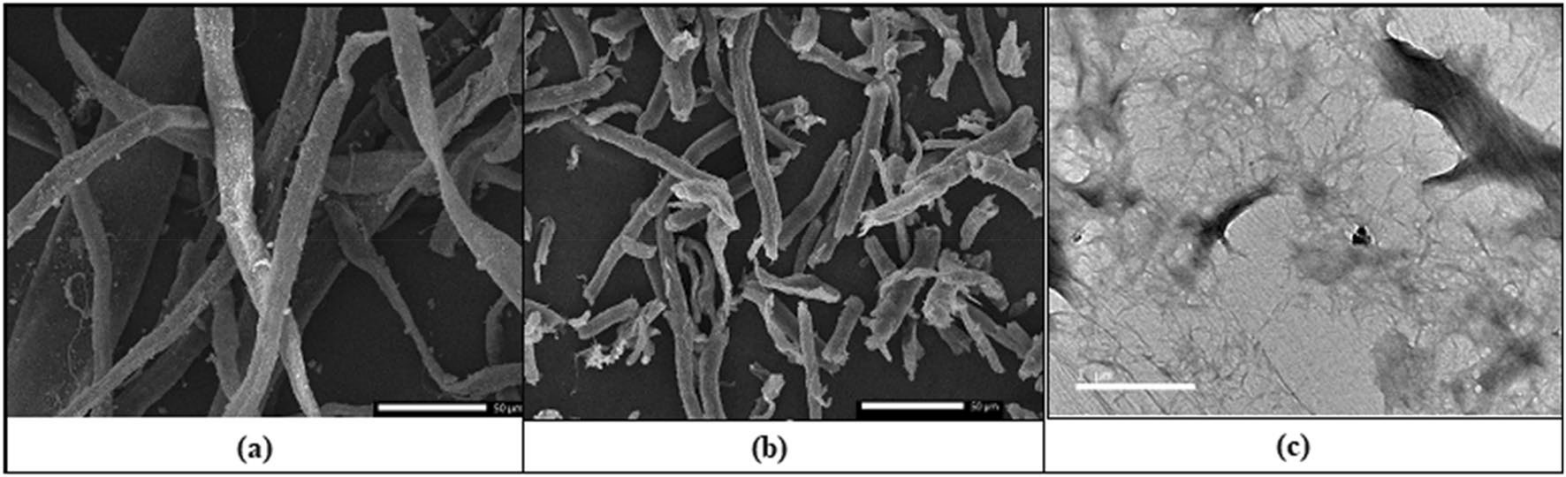
SEM images of (**a**) OWP and (**b**) extracted cellulose and (**c**) TEM image of CNCs.

**Figure 5:**
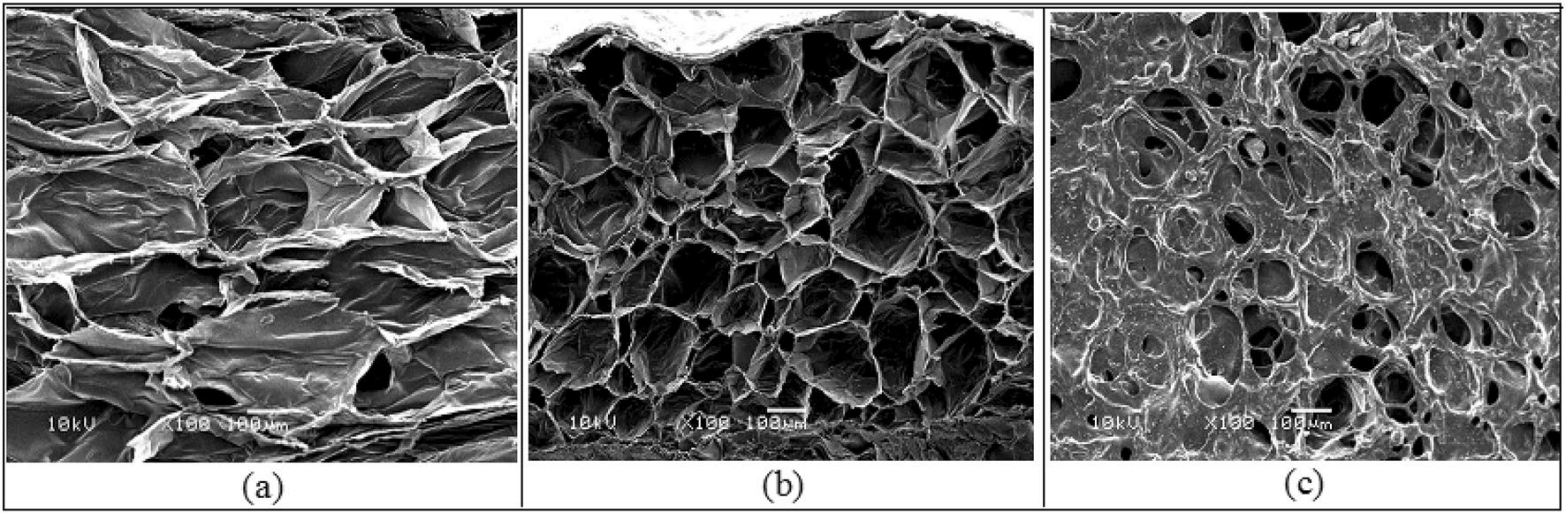
SEM images of the CNCs hydrogels prepared with different CNCs content (**a**) 4% CNCsH, (**b**) 5% CNCsH and (**c**) 6% CNCsH.

Figure 4 shows the SEM images of OWP and extracted cellulose, as well as the TEM image of CNCs. OWP (Fig. 4a) had a diameter of approximately 28–20 µm and a rough surface. The diameter of cellulose (Fig. 4b) became in the range of 9.8–5.7 µm after alkali and bleaching treatment, demonstrating the efficacy of pretreatment processes in eliminating lignin and hemicellulose^44^. In Fig. 4c, the diameter of CNCs has dropped drastically from micrometer to nanometer scale, and they exhibit a rod-like structure with particles ranging in width from 18 to 22 nm and length from 120 to 140 nm^47^. SEM images of CNCsH which were prepared with different CNCs content were shown in Fig. 5. CNCsH display porous inner structure, with a decrease in pore size when CNCs concentration increased that may be the result for formation of more crosslinks which create a narrow pores network structure^41^.

#### Crystallinity analysis

**Figure 6:**
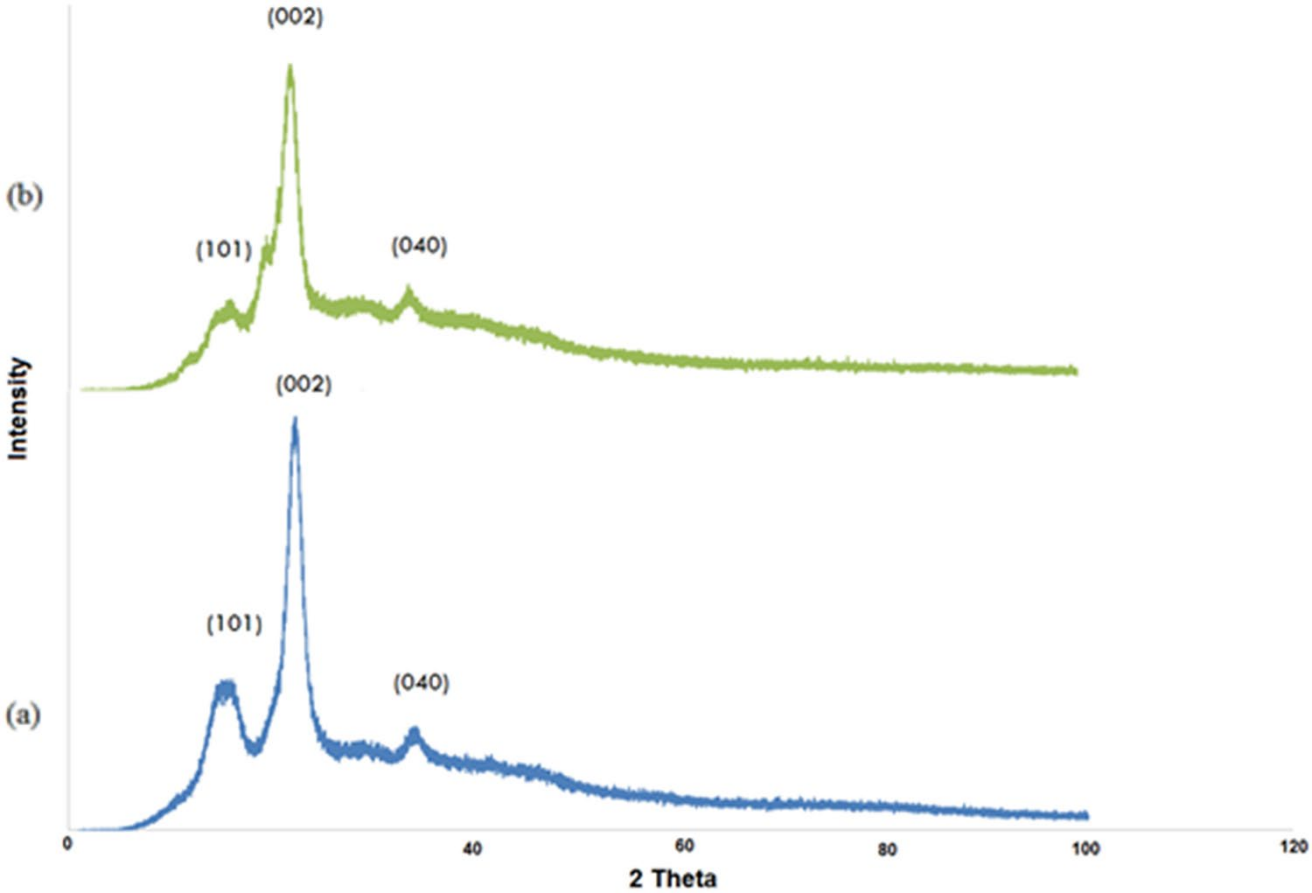
X-ray diffraction patterns of (**a**) OWP, (**b**) extracted cellulose and (**c**) prepared CNCs.

XRD patterns of the extracted cellulose and the prepared CNCs (Fig. 6) showed significant peaks around 2θ = 15°, 22°, and 34° which were assigned to the crystalline planes of (110), (200), and (004), respectively in the crystal structure of cellulose type I allomorph. This result suggested that the crystal integrity of cellulose had been maintained and hydrolysis reaction only had a minor impact on the polymorphism of cellulose I for CNCs sample. The degree of crystallinity (CI) was calculated from Eq. (1) for all the samples, where I_c_ = intensity of peak 2$\theta$ = 22° and I_am_ = intensity of peak 2$\theta$ =15°. The CI increased progressively from (63.7%) for the extracted cellulose to (78%) for the CNCs. The reason for this increase may be because of dissolving of amorphous regions and releasing of crystalline regions during the a acid hydrolysis process, beside the structural changes in alignment to produce a high ordered crystals^47,48^.

### Determination of pH of point of zero charge for the prepared CNCsH (pH _PZC_)

**Figure 7:**
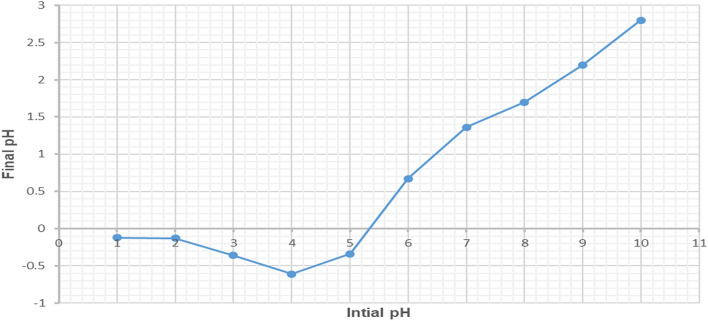
Determination of pH_pzc_ for CNCs hydrogel by the pH drift method.

The change of the initial and final pHs of the CNCsH in NaCl solution was presented in Fig. 7. The pH_pzc_ for the CNCsH, is the point at which adsorbent’s surface is neutrally charged (ΔpH =0), was found to be 5.4. Consequently, the CNCsH surface is positively charged at any pH lower that pH_pzc_ which assumes an increase in the electrostatic attraction between the anionic dye and the hydrogel surface with the decrease in pH, but it became negatively charged at any pH more than pH_pzc_ which decreases the adsorption of negatively charged AR8 dye anions due to electrostatic repulsion force.

### Swelling studies

**Figure 8:**
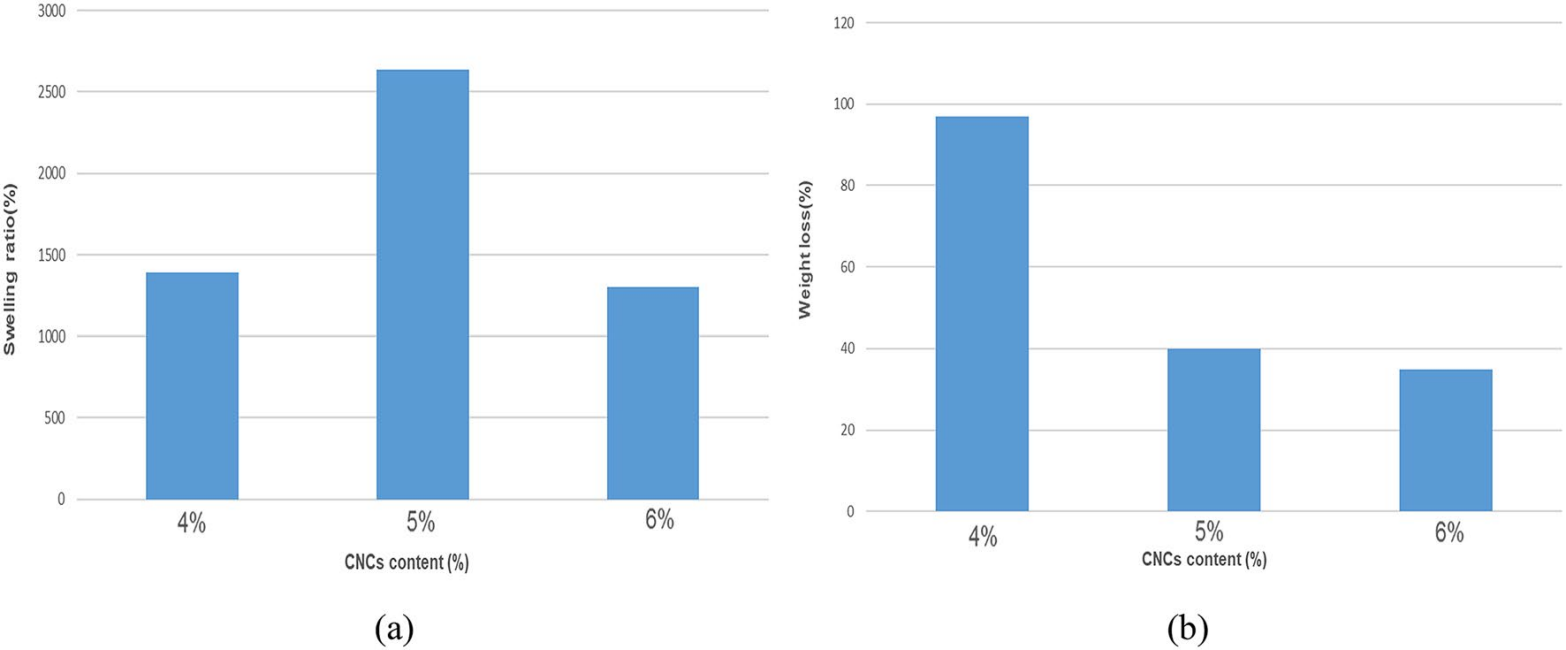
(**a**) Swelling behavior and (**b**) biodegradability study of CNCsH.

Figure 8a illustrates the effect of CNCs content on the swelling capacity of the CNCsH. The swelling ratio was increased with the increase in the CNCs concentration from 4 to 5% from 1800 to 2770%. Then, it was decreased to 1340% with a further increase in CNCs concentration up to 6%. The initial raise in swelling values can be because of the increase in hydrophilicity of CNCsH due to the availability of OH groups, meanwhile the further decrease in swelling ratio can be a result of the decrease in pore size due to the increase of concentration of CNCs that restrict the entry of additional water molecules into its network which was clearly observed in the SEM images^49,50^.

### Biodegradability test

After 10 days of incubation, the weight loss of the CNCs hydrogels was calculated as shown in Fig. 8b indicates that the increase of the content of CNCs, produces a hydrogel more resistant to the attack of microorganisms as the weight loss decreased from 97 to 40% and reaching 35% as the content of CNCs increased from 4 to 5% and 6%, respectively. This may be due to the increase in the crosslinking and the decrease in the pore size, which prevent the imbibition of more soil microorganisms in the swollen hydrogel network^51^.

### Adsorption studies

#### Adsorption mechanism

**Figure 9:**
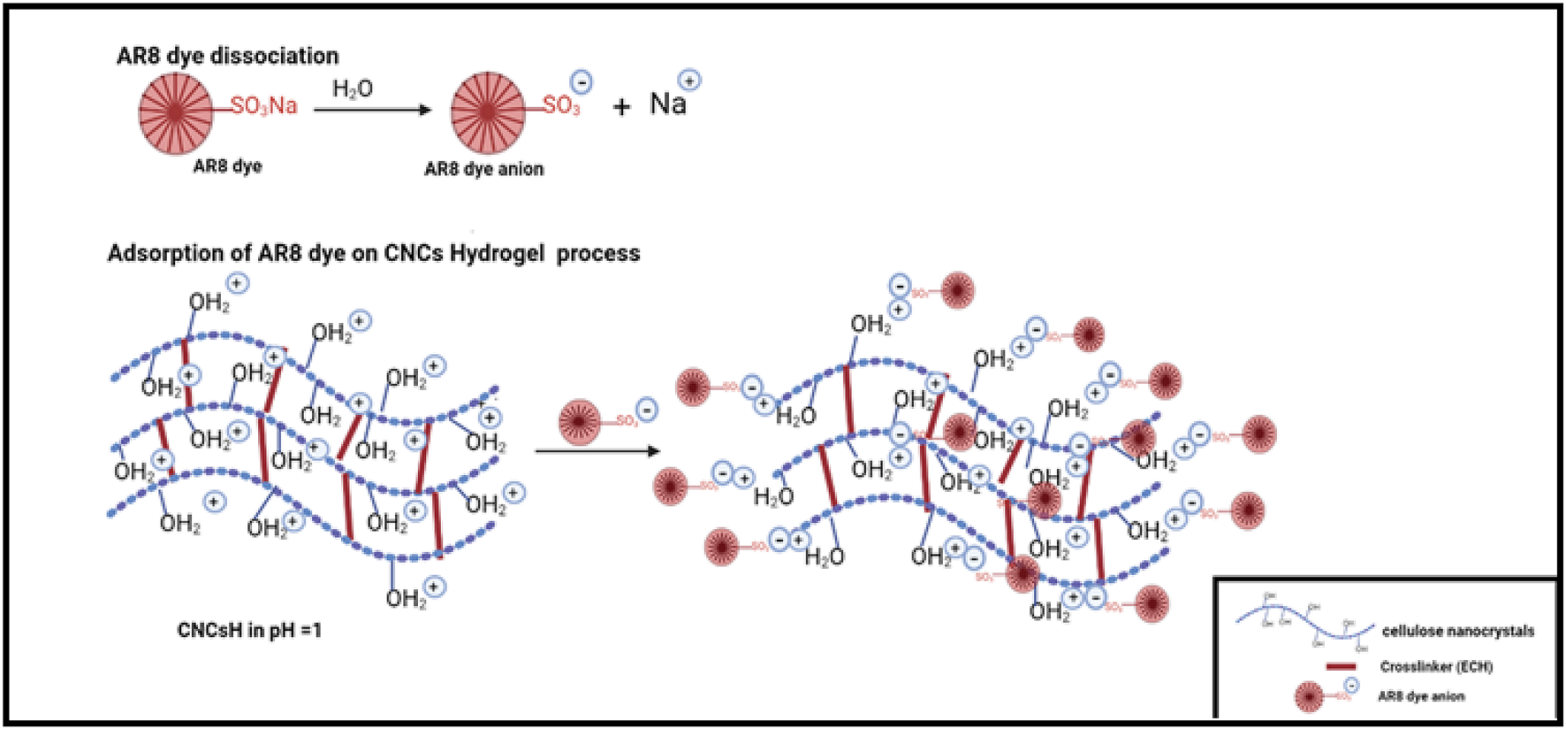
Adsorption of AR8 dye on CNCsH mechanism.

**Figure 10:**
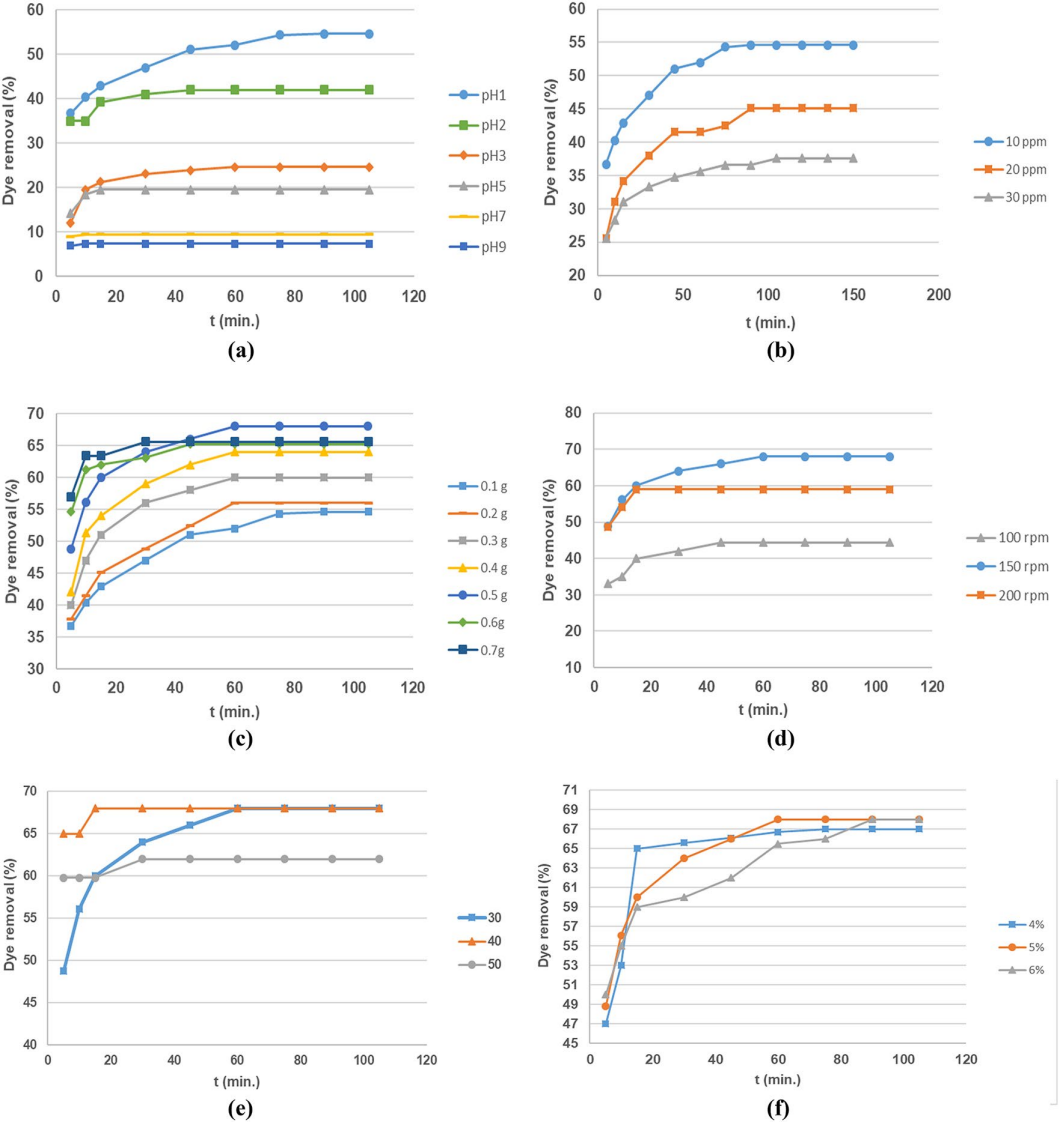
Effects of different factors on the removal percentage of AR8 onto the CNCs hydrogel. (**a**) Effect of pH on the removal percentage of AR8 dye (Initial concentration of AR8dye = 10 ppm, dose = 0.1 g, temp = 30 °C, shaking speed = 150 rpm and CNCs content = 5%). (**b**) Effect of initial concentration of AR8dye on the removal percentage of AR8 dye (pH = 1, dose = 0.1 g, temp = 30 °C, shaking speed = 150 rpm and CNCs content = 5%). (**c**) Effect of dose of hydrogel on the removal percentage of AR8 dye (Initial concentration of AR8dye = 10 ppm, temp = 30 °C, shaking speed = 150 rpm, pH = 1 and CNCs content = 5%). (**d**) Effect of shaking speed of hydrogel on the removal percentage of AR8 dye (Initial concentration of AR8dye = 10 ppm, dose = 0.5 g, temp = 30 °C, pH = 1 and CNCs content = 5%). (**e**) Effect of temperature of hydrogel on the removal percentage of AR8 dye (initial concentration of AR8dye = 10 ppm, shaking speed = 150 rpm, dose = 0.5 g, pH = 1and CNCs content = 5%). (**f**) Effect of CNCs content of hydrogel on the removal percentage of AR8 dye (Initial concentration of AR8dye = 10 ppm, shaking speed = 150 rpm, dose = 0.5 g, pH = 1 and temp = 30 °C).

The mechanism of AR8 dye adsorption process on CNCsH proceeded in two steps as shown in Fig. 9, first AR8 dye which is sulfonate dye(D-SO_3_Na) dissociated into anionic ions (D-SO_3_^−^) and sodium cation (Na^+^) and at pH = 1 aqueous solution, OH groups of the prepared CNCsH are protonated. Secondly, electrostatic attraction take place between the protonated OH group in CNCsH and the anionic ion of the dye^40^. The different factors affecting the removal of the AR8 dye by CNCsH were investigated and presented in Fig. 10.

##### Effect of pH AR8 Adsorption

The pH_pzc_ of the CNCsH, at which the surface of the hydrogel has zero potential charge, was found to be at pH 5.4. At any pH below this value the surface of the hydrogel will have a positive charge, whilst at pH above this the surface will have a negative charge. The effect of pH on the adsorption of AR8 dye by CNCsH was presented in Fig. 10a. It was found that the increase in pH value from 1 to 9 of AR8 solution decreases the removal percentage from 54.6 to 7.4 %. The maximum adsorption occurred at pH 1 where the removal percentage reached 54.6 %. Since AR8 dye is an anionic dye, at acidic pH, the electrostatic attraction force was enhanced between the dye and the CNCsH surface due to the protonation of the OH groups. But, in alkaline conditions, less adsorption of AR8 ions was carried out, this can be due to the presence of excess OH^-^ ions which prevent the protonation of OH groups and cause destabilization of the AR8 ions^52^.

##### Effect of initial dye concentration on AR8 adsorption

The initial concentration of the AR8 dye solution (10, 20 and 30 ppm) showed a significant effect on the adsorption process as illustrated in Fig. 10b. The maximum dye removal was found to be at 10 ppm, with a maximum removal percentage of 54.6%. Conversely, a decrease in the removal percentage reaching 45.1 and 37.6 % was noticed as the initial concentration of dye increased from 20 to 30 ppm, respectively. This can be related to the low ratio of available active sites at hydrogel surface to dye concentration, causing adsorption sites saturation of the hydrogel surface. This in turn indicated a possible formation of a monolayer of dye molecules at the interface with the hydrogel^53^. Moreover, when the concentration of dye increases, there will be electrostatic repulsion between dye molecules which causes competition between dye molecules for the limited CNCsH’s active sites, consequently the dye removal (%) decreased with increasing initial dye concentrations^54^.

##### Effect of hydrogel dosage on AR8 adsorption

By studying the effect of CNCsH dosages on the adsorption process, Fig. 10c, it was found that the removal percentage raised from 56 to 68% as the dosage of the CNCs hydrogel increased from 0.1 to 0.5 g and started to decrease from 68 to 65.6% as the dosage increased from 0.5 to 0.7g, respectively. The results indicate that the CNCsH dosage of 0.5 g is the optimum value for a maximum dye removal. The increase in removal percentage by increasing the dose can be due to availability of more adsorption sites as a result of increasing the hydrogel surface area while the decrease in the removal percentage may attributed to overlapping or aggregation of the hydrogel surface which lead to an increase diffusion path length^55^.

##### Effect of shaking speed on AR8 adsorption

The effect of shaking speed on the adsorption percentage of AR8 dye was elucidated in Fig. 10d. Increasing the shaking speed from 100 to 150 rpm improved the removal percentage from 44.4 to 68% as it enhanced the diffusion of dye molecules towards the surface of the hydrogel by promoting the formation of the external boundary film. Nevertheless, the increase in the shaking speed to 200 rpm reduced the removal percentage to 59%. This may be a result of the excessive contact between the hydrogel surface and dye molecules which caused attrition of the hydrogel and damage its outer surface^56^.

##### Effect of temperature on AR8 adsorption

To study the effect of temperature on the adsorption, the tests were performed at three different temperatures (30, 40, 50 °C). As presented in Fig. 10e, the removal percentage remained constant at 68% when the temperature was increased from 30 to 40 °C. This shows that the removal percentage wasn’t influenced by the increase of temperature from 30 to 40 °C. However, when the temperature rose from 40 to 50 °C the removal percentage decreased to 62%. The increase in temperature led to increasing the mobility of AR8 dye molecules which cause escape of the dye molecules from solid to liquid phase resulting breakage of bonds between the them and active sites of CNCsH and also cause shrinking of CNCsH that pose a decrease in the swelling capacity of CNCsH^57^. This behavior confirms that the adsorption of dyes had an exothermic nature.

##### Effect of different CNCs content on AR8 adsorption

CNCsH's containing different CNCs concentrations were compared regarding their adsorption properties. As represented in Fig. 10f, with the raise of CNCs concentration from 4 to 5%, the removal percentage of AR8 dye increased from 65 to 68%, respectively. But when the CNCs concentration reaches 6 %, the removal percentage decreased to 66%. The reason was as CNCs concentration increased, the pore size of the prepared hydrogel decreased, which made a negative effect on the diffusion of AR8 dye ions into the CNCsH interior surface which consequently impeded the adsorption process^52^.

#### Adsorption isotherm studies

**Figure 11:**
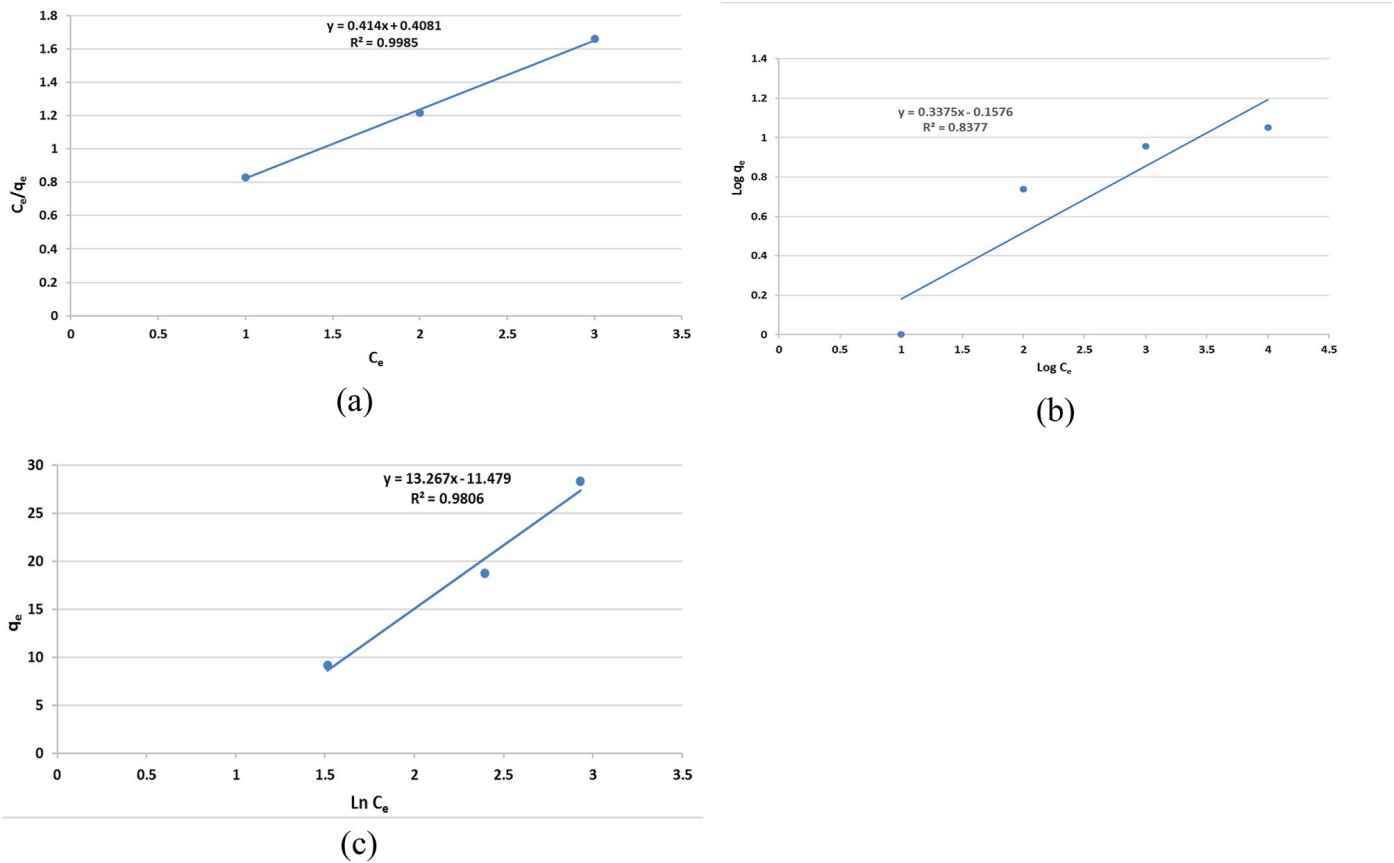
kinetics models (**a**) Langmuir model, (**b**) Freundlich model and (**c**) Tempkin model.

Adsorption isotherm is essentially used to describe how adsorbate interact with adsorbents and it is the key to optimize the use of adsorbents. The adsorption isotherms were investigated for providing information about the adsorption behavior, the possible adsorption mechanism and adsorption capacity. Three models, involving Langmuir, Freundlich and Tempkin models, were employed to describe the isothermal behavior of the adsorption process^58–61^. Three mathematical models are explained in the supplement data S1. The plotting of the $\frac{{\mathrm{C}}_{\mathrm{e}}}{{\mathrm{q}}_{\mathrm{e}}}$ vs C_e_, Log q_e_ vs Log C_e_ and q_e_ vs ln C_e_ are represented in Fig. 11. All the parameters of the studied isothermal models are calculated and summarized in Table 1. By analyzing Table 1 and according to the correlation coefficients (R^2^) values, the experimental results indicated better consistency with Langmuir model as compared to the other two isotherms. The R_L_value was below 1 which indicates that the adsorption was a favorable process. This means that CNCsH surface is primarily composed of homogenous adsorption sites. It also described the monolayer sorption where each AR8 molecule was adsorbed at different localized adsorption sites^62^. The maximum adsorption capacity (Q_o_) (17.12 mg/ g) obtained from Langmuir equation approaches the maximum capacity obtained from the experimental data (11.24 mg/g).

**Table 1:** The different parameters of isotherm models for AR8 removal from solution by CNCsH.

Model	Langmuir				Freundlich			Tempkin		
Parameter	Q_max_	K_L_	R_L_	R^2^	1/n_f_	K_f_	R^2^	B	A	R^2^
	17.12	0.1024	0.25	0.9998	0.3375	1.437	0.8377	13.267	2.375	0.9806

#### Adsorption kinetics studies

Kinetics studies of AR8 dye adsorption on CNCsH can reveal the adsorption mechanism as well as the rate-controlling steps for the adsorption process. Pseudo first order and pseudo second order kinetic models were used to evaluate the experimental data to determine the adsorption mechanism^63–66^. The two models were explained in supplement data S1. Moreover, intra-particle diffusion model was also studied in order to investigate the controlling diffusion mechanism^66^ and it is explained in details in the supplement data S1. The calculated parameters of the models were summarized in Table 2.

**Table 2:** Parameters of kinetic models for AR8 removal by CNCsH.

Dye system	Pseudo first order				Pseudo second order		
	q_e,exp_ (mg/g)	K_1_ (min^-1^)	q_e1_ (mg/g)	R^2^	K_2_ (g/mg. min)	q_e2_ (mg/g)	R^2^
**1. Different concentrations W = 0.1 g, shaking speed = 150 rpm, pH = 1, CNCs content = 5% and temperature = 30 °C**							
C_o_ = 10 mg L^−1^	5.46	0.058	3.27	0.9301	0.04	5.6	0.9986
C_o_ = 20 mg L^−1^	9.02	0.023	3.23	0.8841	0.0289	8.5	0.9995
C_o_ = 30 mg L^−1^	11.28	0.024	3.14	0.9343	0.03	11.2	0.9998
**2. Sorbent dosage Shaking speed = 150 rpm, C**_**o**_** = 10 mg L**^**−1**^** ,pH = 1, CNCs content = 5% and temperature = 30 °C**							
w_o_ = 0.1 g	5.46	0.058	3.27	0.9301	0.038	5.7	0.9986
w_o_ = 0.2 g	2.81	0.067	1.63	0.9309	0.0756	3	0.9987
w_o_ = 0.3 g	1.99	0.063	1.17	0.9739	0.15	2.06	0.9999
w_o_ = 0.4 g	1.61	0.066	1.38	0.9584	0.1856	1.67	0.9998
w_o_ = 0.5 g	1.36	0.067	2.06	0.931	0.29	1.4	0.9999
w_o_ = 0.6 g	1.08	0.069	6.18	0.919	0.947	1.09	0.9999
w_o_ = 0.7 g	0.94	0.059	17.8	0.8065	2	0.94	1
**3. rpm W = 0.5 g, C**_**o**_** = 10 mgL**^**−1**^**, pH = 1, CNCs content = 5% and temperature = 30 °C**							
rpm = 100	0.89	0.032	5.09	0.8219	1.049	0.52	0.9997
rpm = 150	1.36	0.067	2.055	0.931	0.79	1.19	0.9999
rpm = 200	1.18	0.048	26.94	0.5236	1.265	1.4	0.9999
**4. Temperature W = 0.5 g, shaking speed = 150 rpm, C**_**o**_** = 10 mg L**^**−1**^**, CNCs content = 5% and pH = 1**							
T = 30 °C	1.36	0.067	2.06	0.931	0.569	1.4	0.9999
T = 40 °C	1.36	0.032	47.56	0.4595	3.367	1.36	1
T = 50 °C	1.24	0.045	25.8	0.714	2.352	1.25	1

Intra-particle diffusion model	K_int_	C	R^2^				
**Initial dye concentration (ppm)**							
10	0.1775	3.6098	0.88				
20	0.3586	5.2316	0.8757				
30	0.3338	7.7321	0.8841				

**Figure 12:**
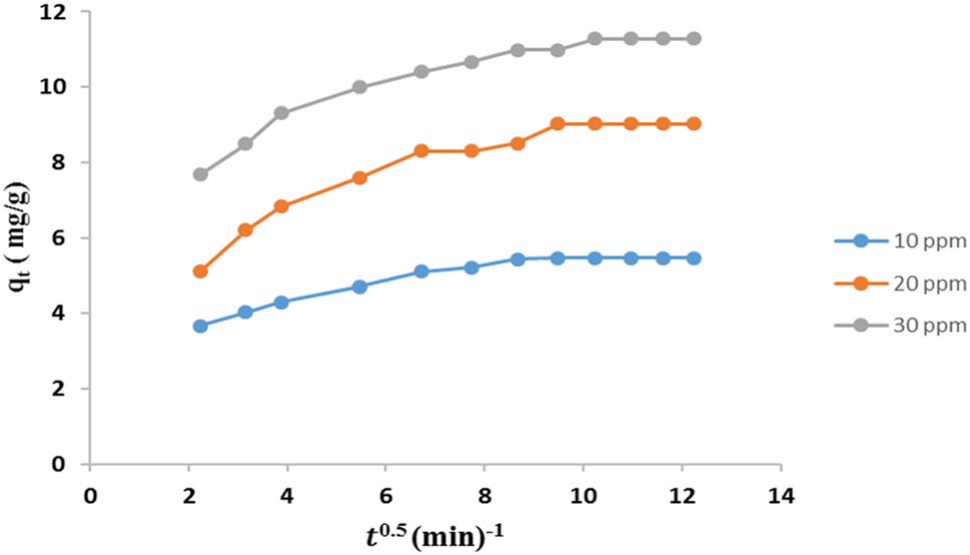
Intra-particle diffusion plots for adsorption at different initial concentration of AR8 on CNCsH.

The pseudo second order model had R^2^ values closer to unity and the values of the theoretical equilibrium adsorption capacity (q_e2_) computed from the pseudo-second-order model were more fitted with the experimental values of equilibrium adsorption capacity (q_e,exp_). So, this prove the superior fit of the AR8 dye adsorption process to the pseudo second order model, confirming that the overall rate is driven by the electrostatic attraction^67^. As represented in Fig. 12, the plots of ${q}_{t}$ vs. ${t}^\frac{1}{2}$ didn't pass through the origin and multi-linear including three linear segments of AR8 dye adsorption was observed. The graph reflects that the intra-particle diffusion is involved in the adsorption process but it isn’t the rate-limiting step and the adsorption of AR8 on CNCsH is governed by another controlling factors. Usually, the first sharper step attributed to the transport of dye molecules from the aqueous solution to the outer surface of the adsorbent by diffusion through the boundary layer. The second step reflects a decrease in the rate of adsorption. While the third step is the diffusion of the dye molecules into the interior small pores of the CNCsH and at this stage, equilibrium was achieved. Value of (C) provide information about the thickness of the boundary layer, the larger is the values the greater is boundary layer diffusion effect. As represented in Table 2, the C value raised with the increase of initial concentration of AR8 which indicate that the increase in initial concentration of dye induce the boundary layer diffusion effect^68,69^.

#### Thermodynamic studies

**Figure 13:**
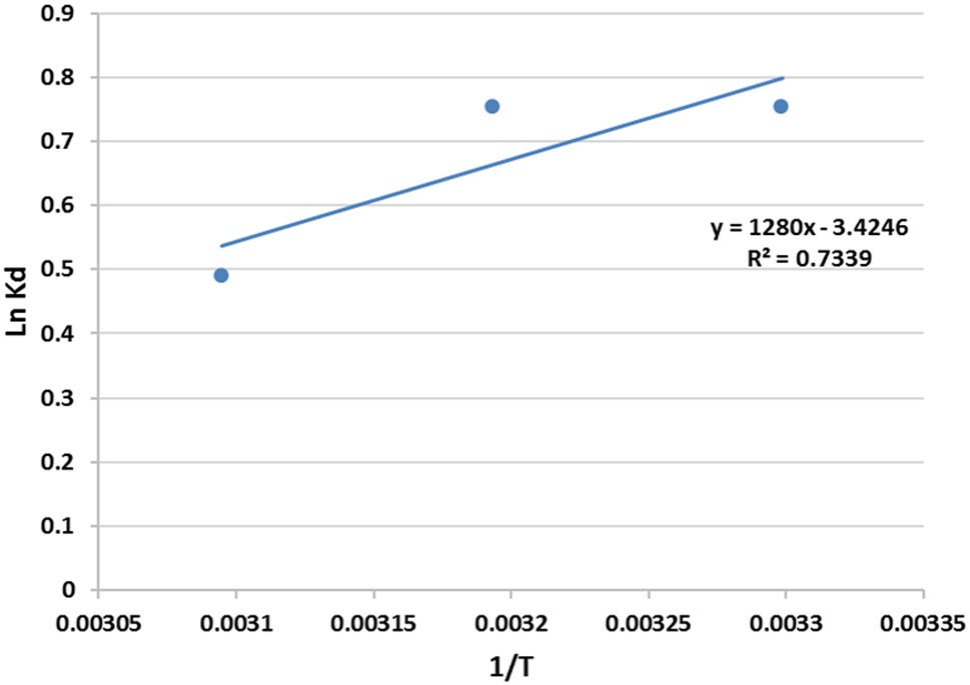
Thermodynamics studies of AR8 dye.

For further evaluation of the feasibility and the effectiveness of temperature on the dye adsorption by CNCsH, thermodynamic parameters including free energy change (∆G°), enthalpy change (∆H°) and entropy change (∆S°) were evaluated by the thermodynamic formulas expressed in following Eqs. (6, 7, 8 and 9)^57,70^.

6 $$\Delta {\text{G}}^{ \circ } = - {\text{RT}}\ln {\text{K}}_{{\text{C}}} { }$$

7 $$K_{C} = \frac{{C_{ads} }}{{C_{e} }}$$

8 $$\ln K_{C} = \frac{{\Delta S^{ \circ } }}{R} - \frac{{\Delta H^{ \circ } }}{RT}$$

9 $$\Delta G^{\circ} = \Delta H^{\circ} - T\Delta S^{\circ}$$

where k_c_ is the thermodynamic constant, C_ads_ is AR8 adsorbate amount (mg/g), C_e_ is the concept of AR8 in the solution at equilibrium (mg/L), R is the universal gas constant (8.314 J/mol. K), and T is the absolute temperature (K). ∆G° is the Gibbs free energy change (kJ/mol), ∆S° is the entropy change kJ/mol. K), and ∆H° is the enthalpy change (kJ/mol). The values of ∆S° and ∆H° were determined based on the intercept and the slope of the plot of (Ln K_c_) against 1/T illustrated in (Fig. 13). All the values were presented in the Table 3. The negative values of ∆G° denoted spontaneous adsorption process. While the negative value of ∆S° indicated the decrease of randomness at the hydrogel/dye interface during the adsorption process. Besides that, the negative value of ∆H° confirms the exothermic nature of the adsorption of AR8 on the CNCsH. Generally, the values of ∆H° gave an indication about the adsorption mechanism. For physical adsorption, the ∆H° is in the range 2–20 kJ/mol, while for chemisorption the ΔHº is in the range of 80–200 kJ/mol. The low value of ∆H° (10.632 kJ/mol) proved that the interaction between the AR8 dye and CNCsH was weak and the adsorption is a more complex physicochemical process same result was observed by Stanciu et al.^71^.

**Table 3:** Thermodynamic parameters of AR8 dye adsorption on CNCsH.

∆H° (Enthalpy) (kJ/mol)	− 10.632	
∆S° (Entropy) (J/mol K)	− 28.45	
∆G° (Gibbs free energy) (kJ/mol)	T °C	
	30 °C	− 19.3
	40 °C	− 19.5
	50 °C	− 19.8

### Reusability study

**Figure 14:**
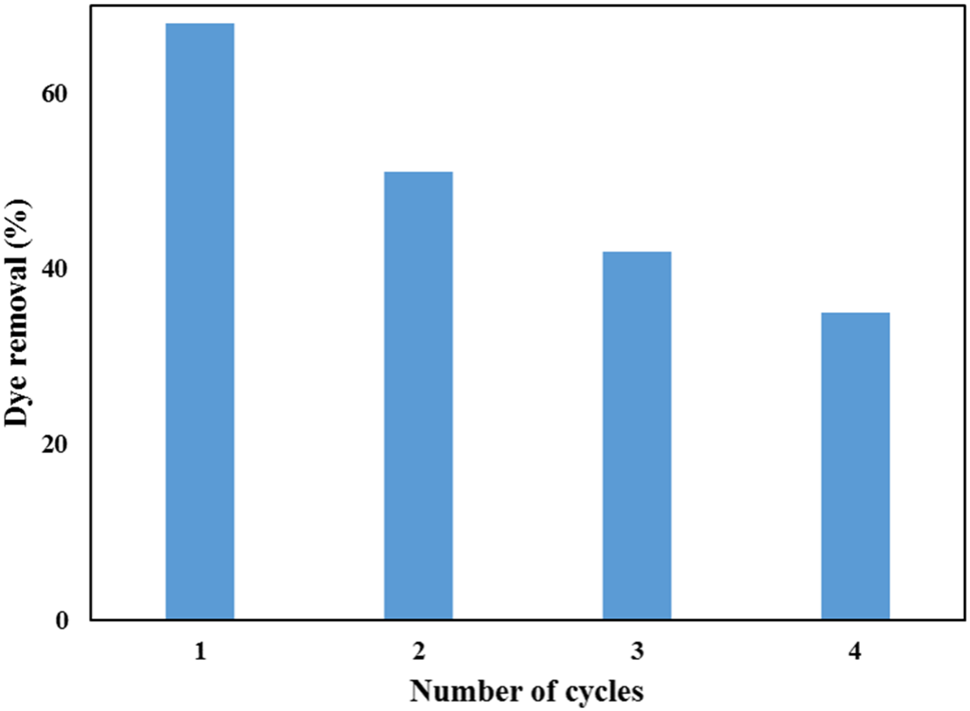
Reusability study of CNCsH for adsorption of AR8 dye.

Figure 14 demonstrates AR8 dye removal percentage on CNCsH after four adsorption-desorption cycles. The dye removal percentage dropped from 68 to 35% after fourth cycles. This is most probably due to degradation of polymer chains which caused by numerous alkali treatment cycles^40^.

### Comparison with nanocrystals based hydrogels

The CNCs-based hydrogel was primarily utilized to remove cationic dyes such methylene blue (MB)^31,72,73^ and heavy metals including. The use of CNCs-based hydrogels to remove anionic dye has been reported in a few papers, the hydrogels were basically prepared by physically or chemically incorporate CNCs or modified CNCs in polymeric network. On the other hand, no research has been done on the creation of CNCs single component hydrogels and their ability to remove anionic dye. Table 4 summarize the maximum adsorption removal efficiencies of same CNCs based hydrogels. Although the maximum adsorption effectiveness of CNCsH is moderate, it can be improved with subsequent modifications.

**Table 4:** Comparison of adsorption capacity of some CNCs based hydrogels.

Hydrogel	Dye	q_e_ (mg/g)	References
2-(dimethylamino) ethyl methacrylate /(amine- and alkyl-modified) nanocrystalline cellulose	Methyl orange	1.64 for pH 1	^74^
		0.95 for pH 10	
Dialdehyde nanocellulose/amphoteric polyvinylamine	Congo red	869.1	^75^
	Acid red GR	1469.7	
	Reactive light yellow	1250.9	
Graphene oxide modified cellulose nanocrystal/poly (N-isopropyl acrylamide) IPN hydrogel	Congo red	728.8	^73^
Cellulose nanocrystals hydrogel	Acid red 8	11.24	This study

## Conclusion

The Biodegradable CNCs hydrogel were successfully fabricated by chemical crosslinking of CNCs, which were prepared from the waste office paper extracted cellulose, in presence of ECH as crosslinking agent. FTIR analysis confirmed the CNCs chemical structure and the crosslinking process. XRD analysis indicated a typical cellulose crystal lattice of type (I) with high crystallinity of (78%). TEM image exhibited that the prepared CNCs were in the range of 18–22 nm in width and from 120 to 140 nm with shape of a rod like structure. The percentage of swelling ratio of the hydrogel increased from 1800 to 2770 % as the concentration of CNCs increased from 4 to 5% as a result of increase of the active function groups but the swelling decreased to 1340 % when the concentration of CNCs raised to 6% due to decrease in pore size which clearly appeared in SEM images. It was found from biodegradability test that the weight loss of the hydrogels decreased from 97 to 40 % and reaching 35% as the content of CNCs increased from 4% to 5% and 6%, respectively that may be due to the decrease in pore size of the hydrogel. The experimental data of the dye adsorption was better fitted to the pseudo second-order and Langmuir isotherm models, indicating that the adsorption of AR8 dye onto the hydrogels was spontaneous, exothermal monolayer adsorption through a physicochemical process with maximum calculated adsorption capacity of (17.12 mg/ g), which is considered close enough to the experimental value of (11.24 mg/g). Finally, this research study had confirmed that CNCs can prepared using simple and low cost method and CNCsH can be used as a low cost and environmentally friendly adsorbent for the removal of AR8 dye from aqueous solution.

## Supplementary Information

Supplementary Information.

## Data Availability

The datasets generated during and/or analyzed during the current study are available from the corresponding author on reasonable request.
