# Supplementary material for: Biodegradable cellulose nanocrystals hydrogels for removal of acid red 8 dye from aqueous solutions
Source: Sci Rep. 2022 Apr 19;12:6424. doi: 10.1038/s41598-022-10087-1 (PMC9019039; doi:10.1038/s41598-022-10087-1)
Supplement: Supplementary file 1 — Supplementary Information. [file 41598_2022_10087_MOESM1_ESM.pdf]

Biodegradable cellulose nanocrystals hydrogels for removal of acid red 8 dye from aqueous solutions

Radwa Mohamed Abdel Aziz[1]. Azza El-Sayed El-Maghraby[2]. Wagih Abdel Alim Sadik[1]. Abdel Ghaffar Maghraby El-Demerdash[1]. Eman Aly Fadl[1].

[1] Department of Materials Science, Institute of Graduate Studies and Research, Alexandria University, 163 Horreya Avenue, Alshatby, Alexandria 21526, Egypt

[2] Fabrication Technology Research Department, Advanced Technology and New Materials Research Institute (ATNMRI), City of Scientific Research and Technological Applications (SRTA-City)

[*]Corresponding author: igsr_radwa.mohamed@alexu.edu.eg

1. Adsorption isotherms

Langmuir model depicts a monolayer adsorption of dyes molecules on definite localized active sites. It also assumes that this active site has equivalent energy and no transmigration of the dye molecules from the hydrogel surfaces [1,2]. The linearized form of Langmuir isotherm is represented in equation (1).

$$\frac{C_e}{q_e} = \frac{1}{K_L Q_o} + \frac{C_e}{Q_o} \quad (1)$$

Where C_e is the equilibrium concentration of adsorbate dye in solution (mg/L), q_e is equilibrium concertation of adsorbed dye in the CNCsH (mg/g), Q_o is the maximum adsorption capacity for forming a monolayer (mg/g) and K_L expresses Langmuir constant (L/mg).

The plotting of the $\frac{C_e}{q_e}$ values in relation to the C_e values, a straight line is obtained with a slope $\frac{1}{Q_o}$ and an intercept of $\frac{1}{K_L Q_o}$ from which Langmuir constants "K_L" and "Q_o" can be calculated . The significant characteristics of Langmuir isotherm are represented in terms of R_L, a dimensionless equilibrium parameter called a separation factor which expressed in equation (2).

$$R_L = \frac{1}{1 + K_L C_o} \quad (2)$$

Where K_L is Langmuir constant and C_o is the initial dye concentration (mg /L). The R_L value is used to describe whether the adsorption process is unfavorable (R_L>1)), favorable (0 <R_L<1), linear (R_L=1) or irreversible (R_L=0).

Freunchlish isotherm assumed that a multi-layer adsorption processes happen on heterogeneous surfaces where the active sites had different energies [3]. The mathematical formulation of this model is expressed in equation (3) as follows:

$$\log q_e = \log K_F + \frac{1}{n_f} \log C_e \quad (3)$$

Where q_e is the amount of is the AR8 dye adsorbed at equilibrium (mg/g), K_f is freundlich

isotherm constant, n is adsorption intensity, C_e is the equilibrium concentration of adsorbate (mg/L). when the values of Log q_e were plotted against Log C_e, a straight line was yielded. $\frac{1}{n_f}$ and K_f were determined from the slope and the intercept, respectively. The value of $\frac{1}{n_f}$ is ranged from 0 to 1 and indicates the surface heterogeneity which increases as the value approaches zero. A value of $\frac{1}{n_f}$ less than 1 denotes a normal Langmuir isotherm, whilst $\frac{1}{n_f}$ greater than 1 denotes cooperative adsorption .

Tempkin model considered the effects of the interaction between the dye molecules and the hydrogel in the adsorption process. It assumes that, as a result of such interactions, the adsorption heat of all molecules in the layer would decrease linearly with the increase in coverage [4]. The model is expressed in equations (3,4) as follows:

$$q_e = \frac{RT}{b} \ln A + \frac{RT}{b} \ln C_e \qquad (3)$$

$$B = \frac{RT}{b} \qquad (4)$$

where A is Tempkin isotherm equilibrium binding constant (L/g), b is the Tempkin isotherm constant, R is the universal gas constant (8.314 J/mol/K), T is the absolute solution temperature (K), and B is the constant related to heat of sorption (J/mol). A and B were calculated from the intercept and the slope, after plotting qe verses ln Ce (Fig.S1c).

2. Adsorption kinetics

When adsorption driving forces are caused by physisorption, the pseudo first order model is used. It is defined using the integrated linear mathematical equation (5) [5,6].

$$\log(q_{e,exp} - q_t) = \log q_{e1} - \frac{k_1}{2.303} t \qquad (5)$$

$q_{e,exp}$ is the experimental equilibrium concentration of the adsorbed dye on the CNCsH (mg/g), q_{e1} is the theoretical equilibrium concentration of the adsorbed dye on the CNCsH (mg/g), q_t is the equilibrium concentration of adsorbed dye at time t (mg/g) and k_1 is the equilibrium rate constant of pseudo-first-order equation (min^{-1}). From the intercept and the slope of the straight line resulting from the plot of log ($q_{e\,exp}$ - q_t) against time (t), The values of q_{e1} and k_1 could be calculated.

While pseudo-second-order usually associated with conception or electrostatic attraction process. It is expressed by equation (6) [7,8].

$$\frac{t}{q_t} = \frac{1}{k_2 q_{e2}^2} + \frac{t}{q_e} \qquad (6)$$

where q_{e2} is the theoretical equilibrium concentration of the adsorbed dye in CNCsH(mg/g) and

k_2 is the equilibrium rate constant of pseudo-second-order equation (g/mg.min). By plotting $\frac{t}{q_t}$ versus time (t) values $\frac{1}{k_2 q_{e2}^2}$ and $\frac{1}{q_e}$ can be deduced from the intercept and the slope of the result straight line.

The intra-particle diffusion studies the possibility of AR8 dye species to diffuse into the interior sites of the particles of adsorbent was tested with Weber-Morris equation[9] which given by equation (7)

$$q_t = K_{int} t^{\frac{1}{2}} + C \quad (7)$$

Where: K_{int} is the intra-particle diffusion rate constant (mg. g $^{-1}$ .min$^{-1/2}$), q_t is the amount of AR8 adsorbed per unit mass of CNCsH and C is the intra- particle diffusion constant .

3. References

1 Foo, K. Y. & Hameed, B. H. Insights into the modeling of adsorption isotherm systems. *Chemical engineering journal* **156**, 2-10, doi:https://doi.org/10.1016/j.cej.2009.09.013. (2010).

2 Dada, A., Olalekan, A., Olatunya, A. & Dada, O. Langmuir, Freundlich, Temkin and Dubinin–Radushkevich isotherms studies of equilibrium sorption of Zn2+ unto phosphoric acid modified rice husk. *IOSR Journal of Applied Chemistry* **3**, 38-45, doi:https://doi.org/10.9790/5736-0313845 . (2012).

3 Kuang, Y., Zhang, X. & Zhou, S. Adsorption of methylene blue in water onto activated carbon by surfactant modification. *Water* **12**, 587, doi:https://doi.org/10.3390/w12020587 (2020).

4 Hameed, B., Tan, I. & Ahmad, A. Adsorption isotherm, kinetic modeling and mechanism of 2, 4, 6-trichlorophenol on coconut husk-based activated carbon. *Chemical engineering journal* **144**, 235-244, doi:https://doi.org/10.1016/j.cej.2008.01.028. (2008).

5 Aly, Z., Graulet, A., Scales, N. & Hanley, T. Removal of aluminium from aqueous solutions using PAN-based adsorbents: characterisation, kinetics, equilibrium and thermodynamic studies. *Environmental Science and Pollution Research* **21**, 3972-3986, doi:https://doi.org/10.1007/s11356-013-2305-6. (2014).

6 NA, T. Magnetic peanut hulls for methylene blue dye removal: isotherm and kinetic study. *Global NEST* **18**, 25-37, doi:https://doi.org/10.30955/gnj.001730 (2015).

7 Qiu, H. *et al.* Critical review in adsorption kinetic models. *Journal of Zhejiang University-Science A* **10**, 716-724, doi:https://doi.org/10.1631/jzus.a0820524 (2009).

8 El-Maghraby, A. & Taha, N. A. Equilibrium and kinetic studies for the removal of cationic dye using banana pith. *Adv. Environ. Res., Int. J* **3**, 217-230, doi:https://doi.org/10.12989/aer.2014.3.3.217 (2014).

9 Al-Musawi, T. J., Mengelizadeh, N., Al Rawi, O. & Balarak, D. Capacity and modeling of acid blue 113 dye adsorption onto chitosan magnetized by Fe2O3 nanoparticles. *Journal of Polymers and the Environment* **30**, 344-359 (2022).
